# PD04 Economic Evaluation Of Neonatal Screening Strategies For Congenital Cytomegalovirus In Spain

**DOI:** 10.1017/S0266462325101426

**Published:** 2025-12-29

**Authors:** Guillem Torres-Pagès, Jessica Ruiz-Baena, M. Pellicer, Ana Alarcón, Maria Dolors Estrada Sabadell, Rosa Maria Vivanco-Hidalgo

## Abstract

**Introduction:**

Congenital cytomegalovirus (CMVc) is a major cause of neonatal morbidity, with long-term effects such as hearing loss and neurodevelopmental impairments. Screening strategies, including systematic and targeted approaches, aim to improve early detection and management. This study evaluated the cost utility of these strategies to inform decision-making in Spain’s healthcare system.

**Methods:**

A cost-utility analysis was performed using a decision tree and Markov model over an 18-year horizon. Incremental cost-effectiveness ratios (ICERs) were calculated based on quality-adjusted life years (QALYs). The analysis compared systematic screening using a saliva polymerase chain reaction test, targeted screening linked to universal newborn hearing screening (UNHS), and symptom-based detection. Sensitivity analyses explored the robustness of results, focusing on key cost and effectiveness variables.

**Results:**

Systematic screening detected 1,547 CMVc cases at birth, compared with 112 cases for targeted screening and 49 cases for symptom-based detection. At 18 years, systematic screening had the lowest number of patients with hearing loss and neurodevelopmental impairments. The ICER for systematic screening was EUR33,225 per QALY, compared with symptom-based detection and EUR37,254 per QALY compared with targeted screening. Targeted screening demonstrated an ICER of EUR6,912 per QALY compared with symptom-based detection.

**Conclusions:**

Targeted screening linked to UNHS is a cost-effective strategy for detecting CMVc in Spain. Systematic screening, while identifying more cases, exceeded cost-effectiveness thresholds and may not be economically viable under current conditions. Optimizing the cost of systematic screening could improve feasibility and should be a focus of future research.

